# Novel functional variants at the GWAS-implicated loci might confer risk to major depressive disorder, bipolar affective disorder and schizophrenia

**DOI:** 10.1186/s12868-018-0414-3

**Published:** 2018-04-19

**Authors:** Leonid O. Bryzgalov, Elena E. Korbolina, Ilja I. Brusentsov, Elena Y. Leberfarb, Natalia P. Bondar, Tatiana I. Merkulova

**Affiliations:** 1 The Federal Research Center Institute of Cytology and Genetics, The Siberian Branch of the Russian Academy of Science, 10 Lavrentyeva Prospekt, Novosibirsk, Russian Federation 630090; 2 The Novosibirsk State University, 1 Pirogova st., Novosibirsk, Russian Federation 630090

**Keywords:** Genetic of cognition, Major depressive disorder, Bipolar affective disorder, Schizophrenia, Autism spectrum disorders, SNPs, Functional variants, Gene regulation

## Abstract

**Background:**

A challenge of understanding the mechanisms underlying cognition including neurodevelopmental and neuropsychiatric disorders is mainly given by the potential severity of cognitive disorders for the quality of life and their prevalence. However, the field has been focused predominantly on protein coding variation until recently. Given the importance of tightly controlled gene expression for normal brain function, the goal of the study was to assess the functional variation including non-coding variation in human genome that is likely to play an important role in cognitive functions. To this end, we organized and utilized available genome-wide datasets from genomic, transcriptomic and association studies into a comprehensive data corpus. We focused on genomic regions that are enriched in regulatory activity—overlapping transcriptional factor binding regions and repurpose our data collection especially for identification of the regulatory SNPs (rSNPs) that showed associations both with allele-specific binding and allele-specific expression. We matched these rSNPs to the nearby and distant targeted genes and then selected the variants that could implicate the etiology of cognitive disorders according to Genome-Wide Association Studies (GWAS). Next, we use DeSeq 2.0 package to test the differences in the expression of the certain targeted genes between the controls and the patients that were diagnosed bipolar affective disorder and schizophrenia. Finally, we assess the potential biological role for identified drivers of cognition using DAVID and GeneMANIA.

**Results:**

As a result, we selected fourteen regulatory SNPs locating within the loci, implicated from GWAS for cognitive disorders with six of the variants unreported previously. Grouping of the targeted genes according to biological functions revealed the involvement of processes such as ‘posttranscriptional regulation of gene expression’, ‘neuron differentiation’, ‘neuron projection development’, ‘regulation of cell cycle process’ and ‘protein catabolic processes’. We identified four rSNP-targeted genes that showed differential expression between patient and control groups depending on brain region: *NRAS*—in schizophrenia cohort, *CDC25B*, *DDX21* and *NUCKS1*—in bipolar disorder cohort.

**Conclusions:**

Overall, our findings are likely to provide the keys for unraveling the mechanisms that underlie cognitive functions including major depressive disorder, bipolar disorder and schizophrenia etiopathogenesis.

**Electronic supplementary material:**

The online version of this article (10.1186/s12868-018-0414-3) contains supplementary material, which is available to authorized users.

## Background

Looking back over the past decade of human genomics, one can therefore assert that the successful completion of Human Genome Project [1] and 1000 genomes [2] pilot project produced a remarkable increase in our knowledge on genetic variants. Among these, the most common type of variation are single nucleotide polymorphisms abbreviated to SNPs. One estimate is that there are 150 million SNPs in the human genome. However, most SNPs lack any functional significance and only a small fraction of base substitutions can have phenotypic manifestations appearing as changes in the amino acid sequence of the resulting protein product, or changes to the level of gene expression [3, 4]. These *functional* SNPs play a vital role with respect to inter individual’s disease susceptibility and drug response [5, 6]. The framework of the Genome-Wide Association Studies, GWAS, [7] has dominated the investigation of the correlation between the phenotype and certain genetic variants. Presently, more than 16,000 SNPs and small insertions/deletions have been associated with specific human outcomes, diseases and traits according to the GWAS Catalog by the US National Human Genome Research Institute (NHGRI) [7, 8]. It should be noted that about 85% of potentially functional variants are expected to be located in non-coding regions, and a smaller number thereof is believed to act through the regulation of gene expression [9, 10].

The problem is it seems impossible to distinguish the association signals detected from a causative variant and from a number of *tag* SNPs that are likely part of a larger region of linkage disequilibrium [11]. Moreover, several causal variants may converge to create the significant GWAS signals, which are related to one common tag SNP [12]. Thus, the association of any genetic variants with the disease does not necessarily mean the functionality of these variants. The GWAS-implicated associations accordingly, can be difficult to transfer into the understanding of molecular mechanisms that underlie the phenotypic outcome. In general, GWAS signals have rarely been tracked to causal polymorphisms thus far. This adds to the complexity of the development of effective methods for disease treatment and prevention [13].

A second problem is the significant heterogeneity of natural human populations that can be taken care of through proper quality control and study setup including extended cohorts of patients and controls. Using the data from The Encyclopedia of DNA Elements, ENCODE, [14] can play an important role in contributing to the latter issue. Since the ENCODE Project was initiated with the aim to find all functional elements in the genome, it has accumulated numerous data on chromatin and transcribed genes obtained from various cell lines and tissues, and based on these, candidate regulatory SNPs may be found. In particular, available ChIP-seq data on allele-specific binding of different transcription factors (TFs) could be considered as a clear sign that the SNPs with regulatory potential are located within the genome regions occupied by these factors [15–17]. ChIP-seq data on allele-specific binding of active chromatin marks can also provide important insights towards the localization of regulatory variants particularly in combination with allele-specific expression profiles from RNA-seq [15, 16]. Thus, the study of allele-specific events of any kind seems very valuable for identifying the functional regulatory consequences of non-coding SNPs. Notably; these allow analyzing the functionality of a significant amount of SNPs utilizing a relatively small amount of experimental datasets [17].

There is overwhelming evidence for the existence of substantial genetic influences on general and specific cognitive abilities, and brain-behaviour relationships in healthy and pathological conditions [18, 19]. Nevertheless, until recently the genetics of cognition was constrained by the lack of information. The neurological mutations with rather severe cognitive effects have been practically prevalent throughout the known variants [20]. The current advances in the identification of genetic variation when integrated with the results of genome-wide expression analyses now allow to investigate the molecular-genetic mechanisms that drive cognition and disorders thereof [21]. Therefore, the goal of this work was to reveal novel drivers of certain human neurodevelopmental and neuropsychiatric traits, and/or disorders including major depression, schizophrenia, bipolar affective disorder and autism spectrum disorders. The methodology applied focuses on adopting genome-wide datasets (ChIP-Seq, ChIA-PET and RNA-Seq data) to find the functional SNP variants in the human genome and unravel the underlying mechanisms that are likely to promote cognitive deficits.

## Results

### Algorithm overview

**Fig. 1:**
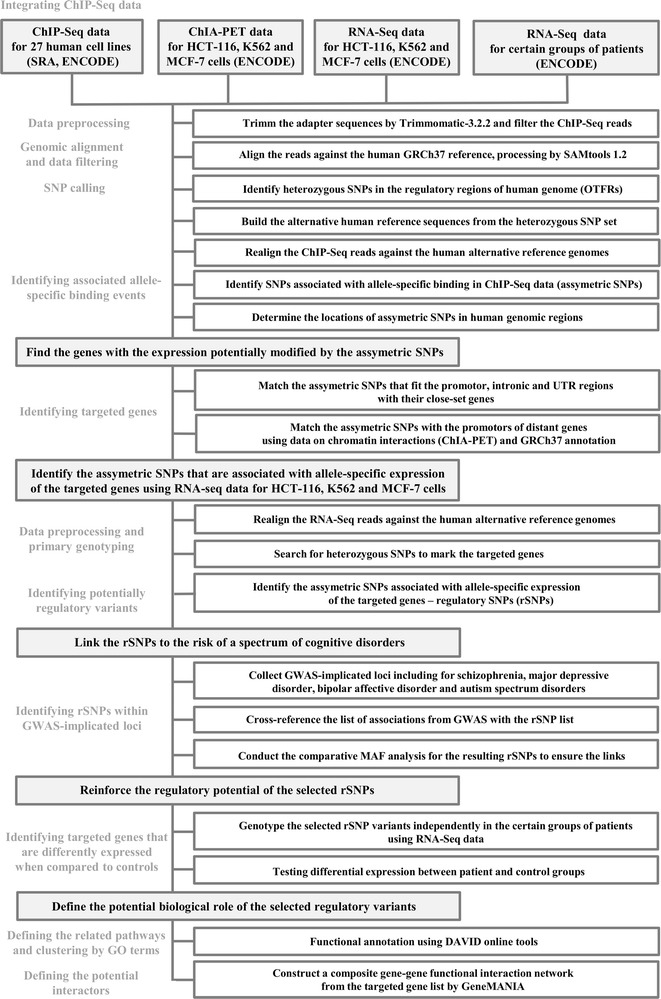
The flowchart representing the graphical overview of bioinformatic pipeline. Light-grey rounded-rectangular shapes present the utilization of raw data. asSNP—SNP that is associated with significant allele-specific binding bias, rSNP—regulatory SNP, DE—differential expression, MAF—minor allele frequency, ENCODE—encyclopedia of DNA elements, ICGC—international cancer genome consortium

An overview of the bioinformatic algorithm is shown in Fig. 1 as a flow chart. Further details regarding each step are described in the “Methods” section

1. *Integrating ChIP*-*seq data from multiple human cell lines.* The motivation was to select functional variants through the comprehensive bioinformatics analysis. At the first stage, we collected and incorporated all ChIP-Seq data for human cell lines of different origin that were available at the time of download (July 2015).
2. *Genomic alignment and data filtering*. The goal of this technical step was to align raw input reads against the human genome. We kept only the hits that passed our primary quality filtering (in particular, alignment coverage) to ensure further accurate identification of the allele-specific events. To avoid the alignment biases that favored the reads containing the reference allele during further bias binding analysis [22] we realigned the ChIP-Seq reads to specific alternative genome sequences at an interim stage.
3. *SNP calling, identifying SNPs in the regulatory regions (OTFRs) from the ChIP*-*Seq data.* Obviously, the search and analysis of functional variants, especially non-coding ones is the major challenging task. In an effort to succeed, the first selection criteria was the location of the heterozygous SNPs within previously defined regulatory genome regions—Overlapping Transcriptional Factor binding Regions, here and further abbreviated to OTFRs [23]. After SNP calling, only polymorphic sites that survived further filtering were analyzed within OTFRs.
4. *Identifying associated allele*-*specific binding events.* At this step, we assessed the representation of different alleles of the selected heterozygous SNPs in the ChIP DNA. The motivation was that the SNPs with a statistically significant allele-specific signal (asymmetric SNPs) could influence the functional activity of the OTFRs in the human genome.
5. *Identifying targeted genes.* We assumed here that the asymmetric SNPs that fit the promotor, intronic and untranslated regions (UTRs) of human genome could directly contribute to the changes in the expression of their nearby genes. The available data on chromatin interactions (ChIA-PET with an RNA pol II antibody) performed for HCT-116, K562 and MCF-7 human cell lines were used in order to determine other possible gene targets that were located distantly from the asymmetric SNP position in the genome.
6. *Identifying potentially regulatory variants (regulatory SNPs, abbreviated here and further to rSNPs).* In this step, we selected the asymmetric SNPs that were associated with significant expression differences of their targeted genes through the analysis of several RNA-Seq datasets: the RNA-Seq data for HCT-116, K562 and MCF-7 cells from ENCODE and human RNA-Seq data from the International Cancer Genome Consortium, ICGC [24]. In the event the identified asymmetric SNPs are associated with significant expression differences of their targeted genes and are found in the population, these effects on the expression can continue in terms of phenotypic differences, including neuropsychiatric traits. To avoid a reference allele mapping bias [16, 25, 26], the RNA-Seq reads were realigned to specific alternative genome sequences. Then the heterozygous markers, namely the heterozygous SNPs mapped in the coding regions of the targeted genes were collected through RNA-Seq data analysis. Next, the significant (p < 0.05) allele-specific expression bias was assessed for the corresponding target genes using the selected markers. The resulting variants were further considered as rSNPs. If there was no SNP ID available for the resulting variant, we provided the designation like *chr10:70716212*, where *chrN* is human chromosome and the latter number—the rSNP position on the chromosome, bp. The targeted genes for the selected rSNP panel (point 5 from the Algorithm list) were recognized as candidate genes that could contribute to phenotypic outcome.
7. *Link the rSNPs to the risk of a spectrum of cognitive disorders.* At this step, we collected GWAS-implicated associations for a spectrum of traits related to cognition and cognitive disorders. Next, we cross-referenced the list of associations from GWAS with the rSNP list. Particularly, we assessed the overlap between the list of rSNPs and the list of the loci from − 10,000 to + 10,000 bp around each GWAS- implicated SNP *index*. Then we specified MAF (minor allele frequencies) values for GWAS indexes and for selected regulatory variants independently through the open-source (dbSNP). We continued with only those previously annotated rSNP variants that had MAF values close to those given by dbSNP for GWAS-implicated indexes. The latter argued the case that the selected rSNPs were closely linked to the GWAS-implicated loci and thus may have a role in cognitive functions and suggest a higher risk of cognitive disabilities or disorders.
8. *Identifying asymmetry in the expression of the targeted genes.* To ensure the regulatory potential of the selected rSNPs we utilized the RNA-Seq datasets for two brain regions: the part of frontal cortex and the part of anterior cingulate from the patients that were diagnosed schizophrenia and bipolar affective disorder available by Xiao et al. [27]. Here we assessed the targeted genes that were differentially expressed between certain patient groups and controls depending on brain region.
9. *Define the potential biological role of the selected regulatory variants.* Further, we assessed the composite functional gene–gene interactions between the targeted genes and the genes most related to the original targeted list by GeneMANIA [28]. We also conducted the gene-annotation enrichment analysis and functional annotation clustering using The Database for Annotation, Visualization and Integrated Discovery, DAVID tools [29, 30].

### Identify SNPs that are associated with allele-specific binding and their targeted genes

Chromatin immunoprecipitation sequencing (ChIP-seq) data of TFs, histone marks and other chromatin-associated factors often need to be interpreted in the context of gene regulation. In the present study, the task required first predicting the allele-specific binding from raw data as a straightforward way to home in on regulatory variation. According to this purpose, SRA ChIP-Seq datasets for 27 human cell lines and samples (Additional file 1) and ENCODE ChIP-Seq datasets for the HCT-116, K562 and MCF-7 cells were similarly analyzed resulting in the identification of 298367 unique heterozygous SNPs within the OTFR regions. Then we selected 14,436 SNPs that were defined as asymmetric—associated with a statistically significant allele-specific signal, and therefore could affect the functional activity of the regulatory regions in the human genome. Next step we analyzed the locations of the asymmetric variants in the human genomic regions and identified their nearby and, in possible cases, distant targeted genes—the genes with the expression that might be modified by the asymmetric SNP variant (see “Methods” section for details). As a result, 12,109 from the totaled analyzed asymmetric SNPs were suggested to affect the expression of 9876 targeted genes and entered further analyses.

### Integrate with gene expression profiling (RNA-Seq) data

We assumed that the allele-specific expression of the targeted genes observed through the RNA-Seq data analysis could be largely attributable to the regulatory impact of the associated polymorphic variants. At this stage, we employed three ENCODE RNA-Seq datasets for HCT-116, K562 and MCF-7 cells. The asymmetric SNPs that were mapped within transcribed genomic regions were also analyzed using the ICGC human RNA-Seq dataset (Methods). Out of 12,109 asymmetric SNPs with allele-specific expression analyzed using heterozygous SNP markers (Methods), 1633 variants (nearly 13%) had evidence of allele-specific expression differences (p < 0.05 by binomial test) of the corresponding targeted genes. These variants were considered rSNPs that were further analyzed as candidate susceptibility factors in cognitive disorders both with their targeted genes (Additional file 2: Table S1).

### Integrate with genome-wide association (GWAS) data

To assess the biological role of identified rSNPs, we examined their overlap with the loci that have been associated with human cognitive disorders or disabilities through GWAS (Methods). For each of resulting 1174 GWAS-implicated index SNPs we examined the presence of identified rSNPs within the − 10,000 to + 10,000 bp window. We assumed here that the certain rSNP is in one linkage group with the index SNP since 1 centimorgan is approximately equal to DNA region of 1 million base pairs [31]. We also examined if these rSNPs have MAF values close to those of GWAS-implicated index SNPs to ensure the linkage (see “Methods” section for details).

**Fig. 2:**
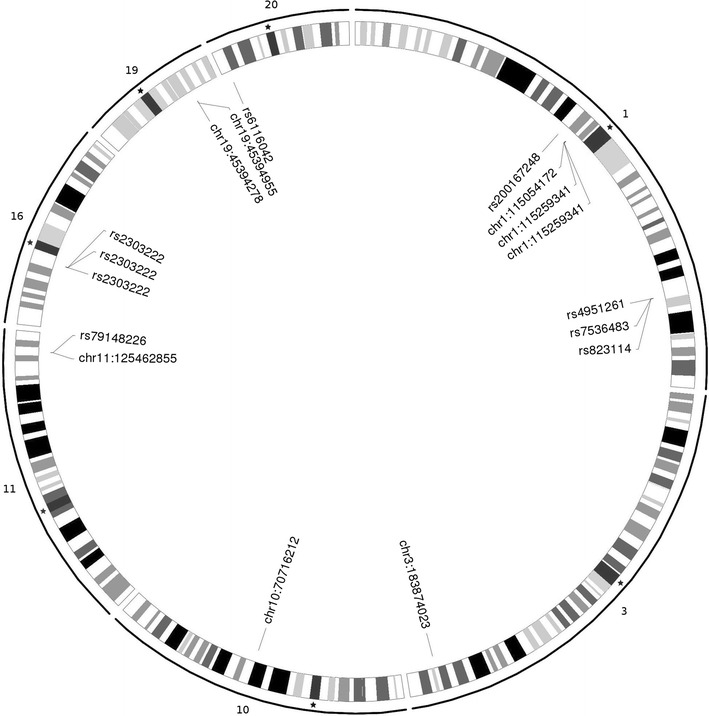
Circular visualization of the distribution of the identified rSNPs in the human genome from R. Grey lines with the numbers along the circumference: individual human chromosomes; the black asterisks: the locations of the centromeres. The IDs for the certain rSNPs are listed in the callouts. The *chr*(chromosome number):*the number*(rSNP position on the chromosome, bp) callouts are given for not-annotated variants (according to dbSNP)

This identified fourteen unique rSNPs within GWAS-implicated loci that were associated with risk of cognitive disorders and totalled twelve targeted genes (Additional file 2: Table S2). These regulatory variants were regarded the candidate disease drivers, including a potential impact on schizophrenia, depression and autism spectrum disorders developing by leading to changes in the expression of corresponding target genes. Figure 2 represents the locations of the selected rSNPs in the human genome.

### Target gene annotation based on gene ontology and biological pathways

**Fig. 3:**
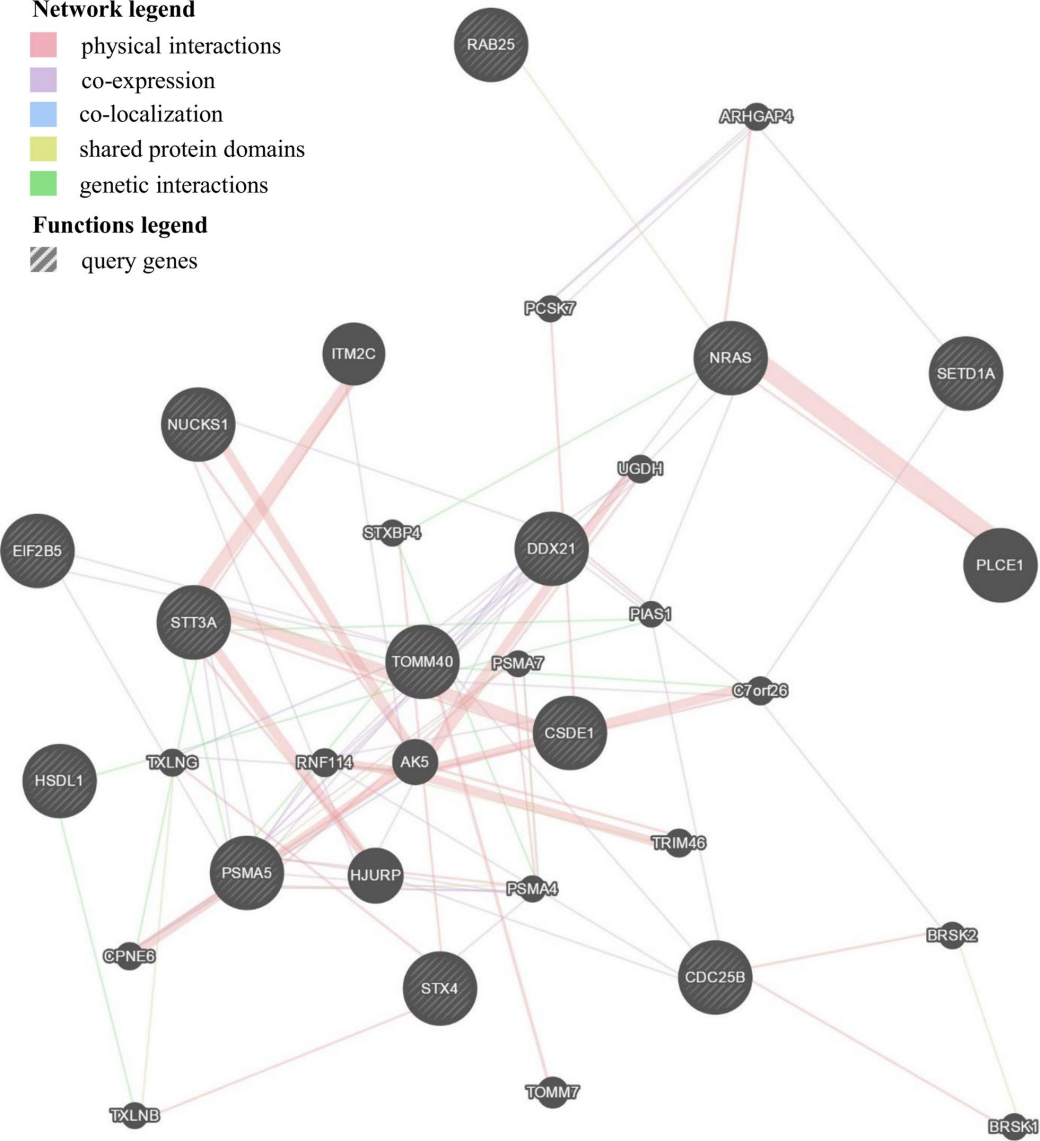
Function prediction of the targeted gene panel by GeneMANIA. The initial list of targeted genes and the type of connections between genes/proteins are illustrated in the functions and networks legends, respectively

Figure 3 shows that physical interactions, co-expression and certain co-localization are apparent among potentially affected genes, such as *TOMM40*, *DDX21*, *NRAS* and *NUCKS1*. Among the targeted genes, the protein products of *NRAS* and *RAB25* have common structural domain and were identified as interacting partners by GeneMANIA [32]. The totaled list of query genes and the interaction gene–gene network details are given in Additional file 2: Tables S3 and S4, respectively.

The functions of the potentially affected proteins together with the other associated proteins (considering physical and genetic interactions by GeneMANIA) were analyzed using a DAVID software. Pathway analyses revealed 15 nominally enriched gene-sets, which showed partial overlap in terms of the underlying genes. The enriched gene-sets included cell cycle, regulation of protein catabolic processes, innate immune response activating cell surface receptor signaling pathway and stimulatory C-type lectin receptor signaling pathway (Additional file 2: Table S5). The results show that these genes are also involved in the positive regulation of protein modification by small protein conjugation or removal; posttranscriptional regulation of gene expression; neuron projection development; cell morphogenesis involved in neuron differentiation and multiply signaling pathways. Thus, in determining the functions of the identified rSNPs in terms of biological behaviors of the potentially targeted genes, we can speculate that they are possibly important in protein metabolism (including catabolism, phosphorylation, transmembrane transport and import into mitochondrial matrix), regulation of cell cycle, regulation of gene expression, neuron differentiation and development. Accordingly, the PSMA5 protein, PSMA4 and PSMA7 proteins that interact with the protein products of the targeted gene panel are associated with the *hsa03050: Proteasome* biological pathway by KEGG (Additional file 2: Table S5). The stimulatory C-type lectin receptor signaling pathway enriched in targeted genes is involved in regulating of immunopathogenesis [33] and guiding the dendritic cells in immunity [34].

### Analysis of effects on human transcriptomes

We further screened a panel of human cases of schizophrenia and bipolar disorder available by Chun Xu and colleagues [27] for expression of rSNP targeted genes when depending on the tissue and the patient cohort.

As a result of analysis by DeSeq 2.0, *NRAS* and *CDC25B* targeted genes happened to be DE in the frontal cortex of schizophrenia-vs-controls and bipolar-disorder–vs-controls groups, respectively when considering a significance threshold of adjusted *P* value ≤ 0.1 after correcting for multiple testing. Both *NRAS* and *CDC25B* genes were higher expressed in the frontal cortex of the certain patient cohorts when compared to controls (logFC of 0,2 and 0,3, respectively). Two other targeted genes happened to be DE in the anterior cingulate of bipolar disorder patients vs controls: *DDX21* (adjusted *P*-value < 0.00012; logFC = −0.7) and *NUCKS1* (adjusted *P*-value < 0.1; logFC = −0.28). No targeted gene was successful to survive the correction for multiple testing in the anterior cingulate of schizophrenia patients vs controls (when considering a significance threshold of adjusted *P*-value ≤ 0.1).

## Discussion

The terms ‘neurodevelopmental and neuropsychiatric disorders’ can cover, to varying degrees, diverse disease classifications including autism, schizophrenia, bipolar disease, etc., that are leading causes of disability worldwide with environmental, genetic and epigenetic risk factors to produce a range of phenotypes in each complex case. Despite seemingly distinct primary diagnoses, the considerable phenotypic heterogeneity, as well as a significant clinical overlap between the subtypes of the certain disease, has been reported [35] as well as an overlap between some genome-wide significant SNPs for different diseases has been observed [36]. The recent data, primarily genome-wide association studies (GWAS), provides the evidence for genetic risk factors. These include genome-wide association with (1) schizophrenia (such as the major histocompatibility complex, MHC, region at 6p22-p21 [37]; (2) depression [38]; (3) bipolar disorder [39], including the variants within *ANK3* [40], *NCAN* [41], *CACNA1C* and *ODZ4* [42] and (4) specific chromosomal regions: 2q, 5, 7q, 15q, 16p [43, 44] and risk genes [45, 46] for autism. Yet, the exact etiology of these disorders remains unknown. It is therefore plausible to believe that different “biological” subgroups of the disease exist, with different underlying genetic etiologies mapping on specific pathways, which underlie specific dysfunctions. Moreover, GWAS often fail to identify replicable common variants [43, 47] or known candidate genes within the GWAS-implicated loci [48, 49]. Assuming that functional alterations affect quantifiable processes including regulatory processes, the comprehensive analysis of genome-wide data is poised to deliver crucial insights into the nature of cognition and the neurodevelopmental and neuropsychiatric disorders thereof.

In this study focusing on the identification of functional risk variants for major neurodevelopmental and neuropsychiatric disorders, we chose to investigate regulatory variation including non-coding, that might strongly influence gene regulation and thus provide relevant information about underlying pathways and molecular mechanisms. Overall, our findings suggested that among the total of identified rSNPs, fourteen fall within the GWAS-implicated loci that are associated with the risk of cognitive disorders (Table 1). Moreover, we identified the corresponding targeted genes that are involved in several processes that seem to be critical for the neurodegeneration and brain dysfunction (Additional file 2: Table S5).

**Table 1:** The rSNPs selected for the GWAS traits related to human diseases and cognitive disorders with their targeted genes

rSNP ID	Targeted gene	GWAS index ID	GWAS-implicated trait
chr10:70716212	*DDX21*	rs2017305	Depression (quantitative trait)
rs200167248	*PSMA5*	rs12049330	Major depressive disorder
chr11:125462855	*STT3A*	rs548181	Combined
chr11:125462855	*STT3A*	rs548181	Schizophrenia
rs79148226	*STT3A*	rs548181	Schizophrenia
rs79148226	*STT3A*	rs548181	Combined
chr1:115054172	*CSDE1*	rs3827735	Autism
chr1:115054172	*CSDE1*	rs11102807	Autism
chr1:115259341	*CSDE1*	rs10489525	Autism
chr1:115259341	*NRAS*	rs10489525	Autism
chr1:115259341	*CSDE1*	rs8453	Autism
chr1:115259341	*NRAS*	rs8453	Autism
rs4951261	*NUCKS1*	rs823114	Parkinson’s disease
rs823114	*NUCKS1*	rs823114	Parkinson’s disease
rs7536483	*NUCKS1*	rs823128	Parkinson’s disease
rs2303222	*SETD1A*	rs11865038	Parkinson’s disease
rs2303222	*AC135050.2*	rs11865038	Parkinson’s disease
rs2303222	*STX4*	rs11865038	Parkinson’s disease
chr19:45394278	*TOMM40*	rs115881343	Cognitive decline (age-related)
chr19:45394278	*TOMM40*	rs2075650	Cognitive decline
chr19:45394955	*TOMM40*	rs115881343	Cognitive decline (age-related)
chr19:45394955	*TOMM40*	rs2075650	Cognitive decline
rs6116042	*CDC25B*	rs3761218	Bipolar disorder
chr3:183874023	*EIF2B5*	rs1969253	Major depressive disorder

The chromosome position in *chr:number* format is given in place of rSNP ID for six of the variants that are not reported in the Database of Single Nucleotide Polymorphisms (dbSNP, Build ID: {138}). Here *chr* is human chromosome and *the number* represents the rSNP position on the chromosome, bp; *GWAS index*—the ID for the GWAS-implicated SNP that is associated with the specific cognitive trait; *combined*: the GWAS-implicated associations for all from the list: autism spectrum disorder, attention deficit-hyperactivity disorder, bipolar disorder, major depressive disorder, and schizophrenia

Overall, recent studies have shown the histone modifications such as phosphorylation, methylation, acetylation, and ubiquitination, can recruit chromatin remodeling protein complexes or alter the structure of chromatin to impact gene expression [50, 51]. In particular, lysine methylation regulates the activation (H3K4, H3K36, and H3K79) as well as repression (H3K9, H3K27, and H4K20) of transcription (reviewed in [52]). The last decade has been marked by an increased interest in relating epigenetic mechanisms to gene transcription, protein synthesis, and synaptic plasticity and distally on learning, memory, complex human behaviors and other cognitive functions [53–57]. In the case of schizophrenia it was shown that a number of genes mapping to risk loci may regulate the gene expression through epigenetic mechanisms [58]. Thus, in terms of potential regulatory consequences, the most interesting result seems to be the identification of *SETD1A* and *DDX21* genes that are targeted by rs2303222 and chr10:70716212 variants in our study. These two rSNPs, rs2303222 and chr10:70716212, were found mapped within the loci from − 10,000 to + 10,000 bp around rs11865038 (GWAS-implicated association for Parkinson’s disease) and rs2017305 (GWAS-implicated association for depression), respectively (Additional file 2: Table S2).

The SET1 family of histone methyltransferases is responsible for depositing the H3K4 methylation mark on promoters of active genes [59, 60]. Particularly, the mutations that modify SETD1A function were documented to contribute to neurodevelopmental disorders, including autism and schizophrenia [61, 62] and also to gene silencing [63]. The *DDX21* gene encodes a nucleolar protein that is a putative RNA helicase characterized by the conserved *DEAD box* motif (Asp-Glu-Ala-Asp). This DDX21 helicase is believed to play important roles in coordinating ribosomal RNA transcription and processing, in RNA editing and RNA transport [64, 65]. Data indicate that DDX21 was confirmed to associate with SET8 methyltransferase [66] and is implicated in a number of human diseases [67]. The SET8 interactor protein specifically catalyzes mono-methylation of K20 on histone H4 (H4K20me1) and thus has been implicated in important processes including gene transcriptional regulation, cell cycle control and maintenance of the genome integrity [68, 69]. Thus, our results make a compelling contribution to the case for the interfaces between regulatory variation and the epigenetic mechanisms to be involved in the pathogenesis of neurodevelopmental and neurodegenerative disorders.

The *DDX21*, rs2303222 targeted gene for RNA helicase, was also shown to be involved in nuclear and mitochondrial splicing. It is worth to note that two other rSNPs—chr19:45394278 and chr19:45394955 novel variants without available SNP ID showed an evidence to contribute to the mitochondria function. These were both associated with cognitive decline according to GWAS and affect the expression of shared *TOMM40* targeted gene, encoding the channel-forming subunit of the TOM translocase complex that is essential for import of protein precursors into mitochondria. Overall, this is in line with the hypothesis that there is an association of autism [70, 71], bipolar disorder [72, 73], schizophrenia and other neuropsychiatric diseases [74–76] with impairments in multiply aspects of mitochondrial function including mitochondrial trafficking that affect neuronal synaptic transmission, neuronal growth and consequently neuronal plasticity and connectivity.

The rs2303222 variant targets one more gene, *STX4,* and our results suggest that this candidate contributes exclusively to Parkinson’s disease. The corresponding protein, syntaxin 4, is involved in synaptic plasticity in hippocampal neurons [77] but has not been previously documented to be associated with Parkinson’s disease, although the synaptic plasticity in the motor cortex was linked to skill learning in mice [78].

Another interesting result regarding all three rs4951261, rs823114 and rs7536483 as rSNPs (regulatory variants) is the shared *NUCKS1* targeted gene encoding a protein that links energy homeostasis, glucose metabolism and transcription. There is an evidence that NUCKS1 can regulate the recruitment of the RNA polymerase II enzyme and the chromatin accessibility in the specific promoter regions [79].

We also recognized shared targeted *STT3A* gene for chr11:125462855 and rs79148226 regulatory variants associated with GWAS-implicated loci for schizophrenia and schizophrenia combined with autism. The significant associations of the variants in *STT3A* locus with the schizophrenia as well as the potential role in pathogenic mechanisms were previously documented [80]. Further, the mutations in *STT3A* are being considered in the differential diagnosis for congenital disorders of N-linked glycosylation (CDG-N-linked) pathway. So it was shown by Freeze and colleagues [81] that the STT3A mutation significantly impairs glycosylation of the biomarker transferrin in a previously unreported case of inherited glycosylation disorder characterized with broad clinical features including microcephaly, cerebellar atrophy, intellectual disability and seizures. The available literature can be used to argue that STT3A functions is important for efficient protein folding and anterograde trafficking [82]. It’s important that the alterations in the proteostasis network including protein folding contribute to abnormal protein aggregation in the pathology of various neurodegenerative diseases [83–85], however the STT3A mutations may directly affect or may not directly affect neurodevelopment and cognitive function.

Summing the evidence, human studies revealed that the brain is particularly sensitive to changes in dosage of various proteins including from regulators to synaptic proteins to the coordinators of the transport and metabolism of brain mRNAs [86, 87]. Dynamic changes that are required for synaptic plasticity, a cellular correlate for learning and memory, rely on protein synthesis and protein degradation. Thus, either of these cellular processes must be finely balanced as significant impairments could result in pathologies.

In our study, novel chr3:183874023 variant within the locus for major depressive disorder was matched to *EIF2B5* gene. This gene encodes a subunit of eukaryotic translation initiation factor 2B (EIF2B), which has a role in protein synthesis as an essential regulator [88]. To give another example, among the nominally enriched targeted gene-sets, the regulation of protein catabolic processes was identified in the present study, in particular through the effects of rs200167248 on *PSMA5* target. This gene was associated with major depressive disorder in the study (Table 1) and encodes a member of the peptidase T1A family, that is a 20S core alpha proteasome subunit [89].

Interestingly, a partial overlap in Gene Ontology terms for the underlying *PSMA5* gene was found here between the ‘protein catabolic processes’ and the ‘innate immune response’ gene-sets. Another targeted gene that was associated with innate immune response is *NRAS* target for chr1:115259341 novel regulatory variant for autism. The protein product for *NRAS* shuttles between the Golgi apparatus and the plasma membrane [90]. This finding may be in correspondence to the evidence that alterations in immune response were recognized among individuals diagnosed with autism spectrum disorders [91, 92]. The chr1:115259341 variant in *NRAS* falls also to the promotor region of *CDSE1* (cold shock domain containing E1) gene. Interestingly, *CDSE1* is a distant target for the chr1:115054172 variant in 5′UTR of *TRIM33*. Both chr1:115259341 and chr1:115054172 rSNPs are located within GWAS-implicated locus for autism (Additional file 2: Table S2). Here our findings are in accordance with the published findings that suggest *NRAS*-*CSDE1* as candidate genes mapping the previously reported linkage region (1p13.2) for autism [93]. CSDE1, also known as UNR (upstream of N-ras), is an RNA-binding protein that may contribute to post-transcriptional control of gene expression in several ways: acting as an activator or inhibitor of translation initiation, stabilizing mRNA or promoting mRNA turnover [94–97]. Thus, the novel regulatory variants of chr1:115259341 and chr1:115054172 are likely to play an important role in the proper control of brain gene expression and, consequently in cognitive functions.

Finally, our findings demonstrate that changes in the expression of a number of candidate genes identified in the study may contribute to the etiopathogenesis of schizophrenia and bipolar disorder. This could be due to the effects of the related rSNPs, regardless of whether the GWAS associations with the certain trait was significant. (In this regard, testing differential expression of the targeted genes between the patients with distinct genotypes appears to provide interesting data. The available datasets have unfortunately not allowed us to conduct the analysis.)

Our data proposed, as mentioned in the “Results” section, that the physical interactions and co-expression do exist among rSNP-targeted genes, including *DDX21* and *NUCKS1*. Interestingly, both these genes were found lower expressed in the anterior cingulate [98] of patients with bipolar disorder when compared to controls. This finding may represent a role of *DDX21* in gene transcriptional regulation [64] and of *NUCKS1* in the DNA damage response [99] as candidate pathways in disease etiopathogenesis. It is worth to note, that the associated rSNPs (Table 1) do not fall in GWAS-implicated loci directly for bipolar disorder. We also could find no evidence of *DDX21* and *NUCKS1* expression changes to bipolar disorder or depression in papers. This is possible because the heterogeneities of disease phenotypes [100, 101].

Further schizophrenia and bipolar disorder patients have shown higher expression of two different targeted genes: *NRAS* and *CDC25B*, respectively, in the frontal cortex than controls. *NRAS*- encoded protein, an intrinsic GTPase of Ras superfamily, is generally associated with cancer advance and progression [102, 103] but is also important in neurodevelopmental disorders for the role to transduce signal from activated receptors further to MAPK cascade [104]. As a proof of concept of candidate pathways, deregulation of CDC25 phosphatase proteins also has an essential role in cell-cycle-driven neuronal death [104]. Moreover, the members of CDC25 family were documented as possible targets that have therapeutic potential in disease [105, 106]. Thus, our findings relating DE targeted genes might lead to new insights to explore the possible links for regulatory variants in different brain regions of schizophrenia and bipolar disorder. However, a role for identified regulatory variants and their gene targets in cognition and disorders thereof is yet to be seen in details.

## Conclusions

Much attention has focused on unravelling the mechanisms by which genetic variation can determine divergence in gene expression levels and, consequently, the phenotypic outcome, yet we are still far from an integrated, evidence-based understanding of the etiopathogenesis of cognitive disorders. Summing up, in the current study, we present novel findings that expand the repertoire of functional variation in human genome, recognize the targeted genes and provide an evidence relevant to disease-associated effects of the identified rSNPs on cognition including on bipolar affective disorder, major depressive disorder and schizophrenia.

## Methods

### Data collection

To investigate the potentially functional SNPs in human genome and further interpret the underlying mechanisms we collected the chromatin immunoprecipitation sequencing (ChIP-Seq, ChIA-PET) and transcriptional profiling (RNA-Seq) data available within the framework of international validated projects such as the Encyclopedia of DNA Elements Project, ENCODE [14] and the International Cancer Genome Consortium, ICGC [24]. In total, we reprocessed 617 ChIP-seq for 29 unique samples and human RNA-seq data sets from two different studies. The samples were from Illumina HiSeq platform. SRA files were converted to fastq format by the fastq-dump tool.

ChIP-seq datasets were performed using antibodies towards histone epigenetic markers (anti-H3K27ac, anti-H3K4 me1, anti-H3K4 me2, anti-H3K4 me3, anti-H3K27 me3), transcriptional factors and a few other chromatin-associated proteins (Additional file 1) and obtained from the following: ENCODE and SRA [107]. ChIA-PET and RNA-Seq datasets for HCT-116, MCF-7 and K562 cells were obtained from ENCODE.

The human RNA-Seq datasets were obtained from the International Cancer Genome Consortium (ICGC) Controlled Data (EGAD00001000215) by Seshagiri et al. [108] and from the NIH Short Read Archive (SRP035524) by Xiao et al. [27].

### Trimming low quality positions

The Trimmomatic-3.2.2 program [109] was applied for the data pre-processing and removing the adapter sequences. To reduce false positives, only the genomic regions that were covered by at least 10 high-quality reads were further analyzed. We also excluded bases with Phred ≤ 20.

### Human reference sequences

We used the human genome build 37 (GRCh37) assembly based on the Genome Reference Consortium Human genome build 37. The genome sequence was downloaded from the UCSC Genome Center [110].

It was shown, that mapping the reads to a single reference genome can significantly affect the outcome of analyses of allele-specificity, both for RNA-seq and ChIP-seq experiments [22, 25]. This will cause the reads representing the reference allele to be preferentially mapped. Thus, in order to avoid mapping bias towards reference alleles we constructed the specific alternative genome sequences by replacing the reference bases at the polymorphic sites with the bases representing the alternate alleles from the collection of all heterozygous SNPs identified directly from ChIP-Seq data. The alternate reference genomes were built independently for the HCT-116, K562 and MCF-7 cell lines. To realign the reads before the search for allele-specific events each specific alternative reference was used independently to utilize the raw data from the corresponding human cell line.

### Data alignment to reference genome sequences

Bowtie2 [111] or TopHAT2 [112] software was applied to map raw paired-end reads to reference genome. Then SAMtools 1.2 [113] and Picard tools were used to discard the duplicated reads and PCR/optical artifacts. We continued with uniquely mapped reads at QMAP > 25 threshold (SAMtools) to reduce the share of sequencing and alignment errors.

### Determination of genomic regions

Our previous analysis of the ChIP-Seq data for multiply original human cell lines resulted in defining a certain set of potentially regulatory regions in the human genome specified as OTFRs [23]. Each of OTFRs (Overlapping Transcriptional Factor binding Regions) showed the associations with specific phenotypic outcome and contained binding sites for two or more transcriptional factors. Thus, to identify functional SNP variants we analyzed only the reads that mapped to OTFRs after realignment of the available raw ChIP-Seq data.

Categories of gene elements, such as intronic regions and 3′\5′ UTRs as well as the transcription start sites for annotated genes (TSSs) were obtained from the GRCh37 annotation data [114]. Promoter regions were set as from 1,8 kbp upstream to 1,8 kbp downstream of all annotated TSSs.

### SNPs calling

After preprocessing and alignment, all the ChIP-Seq reads that mapped within the OTFRs were filtered with the depth of 10 and a mapping quality of 25 set as threshold and then processed using SAMtools pileup, PerlScript and R [115]. As a result, we discovered 298,367 heterozygous SNPs directly from ChIP-Seq data.

### Quality-control metrics for SNPs

To ensure accurate identification of the allele-specific events, all discovered SNPs were subjected to primary filtering. The SNPs in the following categories were eliminated: SNPs within sex chromosomes, mitochondrial DNA and repeat regions [116], SNPs within 5 bp of the regions that map to insertions/deletions, clustered SNPs (that is, those within 10 bp of two other SNPs) and SNPs with significantly different coverage of the reference and alternative alleles (p < 0.05 by binomial test). After this initial quality control, only the reads mapped to the heterozygous sites with at least three alleles that were identified from at least at two samples and two reference genome sequences were further analysed to avoid somatic mutations.

### Analysis of the allele-specific binding events

After an alignment to the alternative genomes described above by Bowtie2 [111], ChIP-Seq reads that were specifically mapped to the specific allele of each heterozygous SNP were counted using SAMtools Perl library. The significant (p < 0.01) differences for read counts between the reference and the alternative alleles were assessed using a two-sided binomial test (implemented in R). The resulting p-values were adjusted for multiple testing by Benjamini–Hochberg adjustment.

### Determination of the targeted genes

To predict the potential targeted (affected) genes nearby the rSNP position we considered that the rSNPs that fall into the intronic, 3′\5′ UTRs and promoter regions may affect the expression specifically of these genes.

We also considered the possibility that each rSNP may affect a promotor of a distant gene, may be outside the associated risk region. To identify such distantly affected genes, we took advantage of an analysis of the recently available ChIA-PET data (“Data collection” section). A minimum of 20 paired ChIA-PET mapped reads that mapped to the certain genomic region was required. The filtering by at least 10 ChIA-PET reads mapped to the genomic regions in both directions was applied to minimize the mapping errors. Next, the average combined area of ChIP-Seq RNA Pol II peaks was calculated in order to determine the effective size of the human genome. Then we built the contact matrix for the regions of ± 1000 bp from the positions of the SNPs associated with allele-specific binding bias and promoter regions of known human genes. The contacts that fit the intersecting and genomic regions and the interchromosomal contacts were excluded from the analysis. Pearson’s agreement criterion (p < 0.001) was applied to assess the reliable contacts.

### Definition of heterozygous SNP markers through RNA-Seq data analysis

RNA sequencing enables defining the allele-specific expression by measuring the sequence reads that are unambiguously mapped to each of the two gene alleles and, accordingly, assessing the preferential expression of the certain allele in a diploid genome. In this case, at least one exonic heterozygous SNP must fit the usable RNA-Seq reads. The asymmetric SNPs that fell in the gene promotors and UTRs and were discovered from RNA-Seq data were directly used for allele-specific expression analysis. Alternatively, we discovered the heterozygous SNP markers within the coding regions of the analyzed targeted genes through the analysis of the ICGC human RNA-Seq dataset (EGAD00001000215).

### Analysis of the allele-specific expression events

RNA reads were realigned to each of the used human reference genomes using TopHAT2 [112]. From the mRNA sequence reads and the location of each assymetric SNPs, the appropriate base read at the location of each polymorphic site was extracted. Then both SAMtools pileup and custom-made R scripts were used to extract allele counts for each SNP. A minimum of 10 RNA reads crossing the heterozygous SNP position was required. An exon was considered to have allele-specific expression if the proportion of the expression between two alleles was significantly greater than 1.5 or less than 1/1.5 (*P*-value ≤ 0.05). The Fisher exact test was used to examine the significance. The resulting p-values were adjusted for multiple testing by Benjamini-Hochberg adjustment.

### Collecting the GWAS-implicated associations

We used the ‘Alzheimer’s disease’, ‘autism’, ‘autism spectrum disorder’, ‘antipsychotic’, ‘anxiety’, ‘bipolar disorder’, ‘cognitive’, ‘depression’, ‘depressive disorder’, ‘Parkinson’s disease’, ‘posttraumatic’ and ‘schizophrenia’ signatures for the GWAS Catalog query to define GWAS-implicated loci that could be related to cognitive disorders.

### Determination of closely linked SNPs

The threshold for difference in the minor allele frequencies (MAF) was set ≤ 15% for considering that two analyzed SNPs fall within one linkage group. These served to choose the linked GWAS-implicated variants for the identified rSNPs when integrating with genome-wide association (GWAS) data. Here the variants, that were reported by the Database of Single Nucleotide Polymorphisms, dbSNP [117] were subjected to a direct allele frequency analysis.

### Functional annotation of targeted genes

The tools of the DAVID Bioinformatics Resources (the Database for Annotation, Visualization and Integrated Discovery, DAVID) [29] were used to provide functional interpretation of targeted gene list derived from the study. All remaining parameters were kept at their default values. The results of functional enrichment analysis are given in the Additional file 2: Table S5.

GeneMANIA [32] web interface was also used to identify direct (physical binding) and indirect (functional) interacting partners of targeted genes based on genomic and co-expression data as well as published experimental data. The input was a list of twelve targeted genes (Table 1) which was then extended by GeneMANIA. Data sets were collected from publicly available databases according to the pipeline described in detail in [118]. A resulting functional association network illustrating the relationships among the genes is presented on the Fig. 3 (see Additional file 2: Tables S3, S4 for details).

### Differential gene expression (DEG) analysis

Once the target genes are predicted, investigating gene expression levels in the case of disease is useful as well to assess the effects of multiple genetic variants on gene function. The RNA-Seq data for the patients from the analyzed cohorts (see “Data collection” section) were realigned to the human reference genomes at an intermediate stage. Then the DeSeq2 Bioconductor package [119] was applied to the data on certain tissues from the patient groups and controls. The resulting p-values were adjusted for multiple testing by Benjamini–Hochberg adjustment. Genes with Benjamini–Hochberg adjusted p-value < 0.01 were considered as significant.

### R code

All statistics and circos imaging was done in R, version 3.1.0 [115]. The custom-made scripts that were applied to perform the analyses and generate the plots are available upon request.

## Additional files

**Additional file 1.** Contains the accession list 1 with the accession numbers for utilized ChIP-Seq datasets (by NCBI archive).

**Additional file 2.** Contains the Tables S1–S5, as listed below: **Table S1.** (The identified rSNPs) contains the IDs and information on the totalled regulatory variants identified in the study. **Table S2.** (The rSNPs that were found associated with cognitive disorders) contains the data on the identified regulatory variants that fell within the − 10,000 to + 10,000 bp window around GWAS-implicated SNPs for analyzed traits related to cognition and cognitive disorders. **Table S3.** (The list of genes in GeneMANIA query) contains the totalled annotation of the targeted genes and their interactors by GeneMANIA. **Table S4.** (The interactions within the targeted gene list by GeneMANIA) contains the information on the composite functional gene–gene interactions between the targeted genes and the genes most related to the original targeted list by GeneMANIA. **Table S5.** (Functional annotation of targeted genes) contains DAVID results for targeted genes and their potential interaction partners by GeneMANIA.

## Abbreviations

SNP(s): single nucleotide polymorphism(s)
rSNP: regulatory SNP
TF: transcriptional factor
OTFR: overlapping transcriptional factor binding region
UTR(s): untranslated region(s)
TSS(s): transcription start site(s)
ChIP-Seq: chromatin immunoprecipitation followed by high-throughput DNA sequencing
RNA-Seq: RNA sequencing
ChIA-PET: chromatin interaction analysis by paired-end tag sequencing
DE (G): differentially expressed (differential gene expression)
MAF: global minor allele frequency
kbp: kilobase pairs
